# Supplementary material for: Investigating healthcare worker mobility and patient contacts within a UK hospital during the COVID-19 pandemic
Source: Commun Med (Lond). 2022 Dec 23;2:165. doi: 10.1038/s43856-022-00229-x (PMC9782286; doi:10.1038/s43856-022-00229-x)
Supplement: Supplementary file 1 — Description of Additional Supplementary Files [file 43856_2022_229_MOESM1_ESM.pdf]

Description of Additional Supplementary Files

File Name: Supplementary Data 1

Description: Aggregated data used in Figures 1, 3 and 5.
