# Supplementary material for: Investigating healthcare worker mobility and patient contacts within a UK hospital during the COVID-19 pandemic
Source: Commun Med (Lond). 2022 Dec 23;2:165. doi: 10.1038/s43856-022-00229-x (PMC9782286; doi:10.1038/s43856-022-00229-x)
Supplement: Supplementary file 3 — Supplementary Material [file 43856_2022_229_MOESM3_ESM.pdf]

Supplementary material

Investigating healthcare worker mobility and patient contacts within a UK hospital during the COVID-19 pandemic

Jared K Wilson-Aggarwal[1], Nick Gotts[1], Wai Keong Wong[2], Chris Liddington[2], Simon Knight[2], Moira J Spyer[3,4], Catherine Houlihan[3,4], Eleni Nastouli[3,4]†, Ed Manley[1]*

*Corresponding author

†Eleni Nastouli on behalf of the SAFER investigators

[1] School of Geography, University of Leeds, Woodhouse, Leeds LS2 9JT, UK

[2]Department of Digital Healthcare Technology, University College London Hospitals NHS Foundation Trust, London, UK

[3]Department of Infection, Immunity and Inflammation, UCL GOS Institute of Child Health University College London, London, UK

[4] Department of Clinical Virology, University College London Hospitals NHS Foundation Trust, London, UK

Key words: nosocomial infections, healthcare-associated infections, healthcare workers, COVID-19, mobility, patient contacts, infection control, behaviour change

Results

Table S1. Difference in the rates of healthcare worker mobility and patient contacts between COVID-19 and non COVID-19 floors in the Tower building at University College London Hospital during the first year of the COVID-19 pandemic. Model estimates & 95% confidence intervals are reported for the percentage change in the daily rate of HCW mobility (N_t^M) and patient contacts (N_t^C) on COVID-19 floors compared to non COVID-19 floors during different stages of the pandemic (first wave, summer lull and second wave).

	Mobility	Patient contacts
First wave	24%*<br>(2%, 45%)	25%*<br>(4%, 46%)
Summer lull	19%<br>(-3%, 41%)	-2%<br>(-23%, 19%)
Second wave	38%***<br>(16%, 60%)	34%**<br>(13%, 55%)

*p < 0.05; **p < 0.01; ***p < 0.001

Table S2. Changes in the rate of healthcare worker mobility and patient contacts on non COVID-19 floors in the Tower building at University College London Hospital during the first year of the COVID-19 pandemic. The mean daily rates and 95% confidence intervals for healthcare worker mobility and patient contacts are reported for the pre-pandemic (baseline). Model estimates & 95% confidence intervals are reported for the normalized difference to baseline for daily rates of healthcare worker mobility (N_t^M) and patient contacts (N_t^C) during each stage of the pandemic (First wave, Summer lull and Second wave). Non COVD-19 floors are those that did not handle the majority of COVID-19 patients (<15%) during the pandemic.

	Mobility				Patient contacts			
Floor	Baseline	First wave	Summer lull	Second wave	Baseline	First wave	Summer lull	Second wave
Imaging (Floor -1)	2.4<br>(2.3, 2.5)	7%<br>(-3%, 18%)	-3%<br>(-15%, 8%)	11%*<br>(0%, 21%)	6.4<br>(5.2, 7.6)	-29%**<br>(-52%, -6%)	7%<br>(-17%, 32%)	-11%<br>(-34%, 11%)
ED (Floor 0)	8.6<br>(8.3, 8.9)	19%***<br>(8%, 29%)	3%<br>(-9%, 14%)	9%<br>(-2%, 19%)	12.7<br>(11.8, 13.6)	20%<br>(-4%, 43%)	30%**<br>(6%, 55%)	-68%***<br>(-90%, -45%)
Day Surgery (Floor 2)	3.1<br>(3, 3.2)	17%***<br>(6%, 28%)	-6%<br>(-18%, 5%)	1%<br>(-9%, 12%)	18.8<br>(15.5, 22.1)	-4%<br>(-27%, 19%)	30%**<br>(6%, 55%)	44%***<br>(22%, 67%)
Nuclear Medicine (Floor 5)	13.4<br>(12.2, 14.5)	-8%<br>(-19%, 3%)	-9%<br>(-20%, 2%)	-16%***<br>(-26%, -5%)	3.8<br>(2.5, 5.2)	-45%***<br>(-69%, -22%)	-12%<br>(-36%, 12%)	4%<br>(-19%, 26%)
Short stay surgery/Gynae (Floor 6)	4.9<br>(4.6, 5.1)	8%<br>(-3%, 19%)	58%***<br>(47%, 69%)	-3%<br>(-13%, 8%)	21.9<br>(21.2, 22.5)	26%*<br>(3%, 49%)	42%***<br>(18%, 67%)	20%<br>(-3%, 42%)
Paediatrics (Floor 11)	7.6<br>(7.4, 7.8)	17%***<br>(6%, 28%)	13%*<br>(2%, 24%)	25%***<br>(15%, 36%)	14.2<br>(13.4, 14.9)	33%**<br>(10%, 56%)	26%*<br>(1%, 50%)	28%**<br>(6%, 51%)
Adolescent (Floor 12)	4.5<br>(4.3, 4.6)	53%***<br>(42%, 64%)	17%***<br>(5%, 28%)	0%<br>(-11%, 10%)	19.7<br>(18.8, 20.6)	-30%**<br>(-53%, -7%)	34%**<br>(10%, 59%)	44%***<br>(21%, 66%)
Oncology (Floor 13)	3.3<br>(3.2, 3.4)	11%*<br>(0%, 22%)	22%***<br>(10%, 33%)	55%***<br>(45%, 66%)	22.1<br>(21.4, 22.9)	24%*<br>(1%, 47%)	28%*<br>(3%, 52%)	28%**<br>(5%, 50%)
Head & Neck (Floor 14)	4.7<br>(4.5, 4.9)	0%<br>(-10%, 11%)	8%<br>(-3%, 20%)	-8%<br>(-18%, 3%)	23.3<br>(22.4, 24.3)	21%<br>(-3%, 44%)	31%**<br>(7%, 56%)	27%**<br>(5%, 50%)
Private wards (Floor 15)	9.3<br>(8.7, 9.8)	-13%**<br>(-24%, -2%)	-26%***<br>(-37%, -14%)	-21%***<br>(-31%, -10%)	9.4<br>(8.3, 10.5)	-41%***<br>(-64%, -18%)	-31%**<br>(-55%, -6%)	51%***<br>(29%, 74%)
Haematology (Floor 16)	7.0<br>(6.7, 7.3)	-7%<br>(-18%, 4%)	-1%<br>(-12%, 11%)	-1%<br>(-12%, 9%)	30.5<br>(29.4, 31.7)	12%<br>(-11%, 35%)	16%<br>(-8%, 41%)	11%<br>(-12%, 33%)

*p < 0.05; **p < 0.01; ***p < 0.001

Table S3. The relationship between the number of COVID-19 patients and the change in HCW mobility and patient contacts on non COVID-19 floors in the Tower building at University College London Hospital during the first year of the COVID-19 pandemic. Model estimates & 95% confidence intervals are reported for the change in normalized difference to baseline for daily rates of healthcare worker mobility (N_t^M) and patient contacts (N_t^C) with every doubling in the total number of COVID-19 patients in the hospital during each stage of the pandemic (First wave, Summer lull and Second wave). Non COVD-19 floors are those that did not handle the majority of COVID-19 patients (<15%) during the pandemic.

	Mobility			Patient contacts		
Floor	First wave	Summer lull	Second wave	First wave	Summer lull	Second wave
Imaging (Floor -1)	6%*<br>(1%, 10%)	-3%<br>(-13%, 6%)	5%*<br>(0%, 9%)	-17%***<br>(-26%, -7%)	9%<br>(-11%, 29%)	-13%**<br>(-23%, -4%)
ED (Floor 0)	12%***<br>(7%, 16%)	6%<br>(-4%, 15%)	2%<br>(-3%, 6%)	-11%*<br>(-20%, -1%)	-2%<br>(-23%, 18%)	14%**<br>(4%, 23%)
Day Surgery (Floor 2)	18%***<br>(13%, 22%)	-6%<br>(-15%, 3%)	-7%**<br>(-12%, -3%)	-10%<br>(-19%, 0%)	-4%<br>(-24%, 16%)	4%<br>(-6%, 13%)
Nuclear Medicine (Floor 5)	1%<br>(-3%, 6%)	8%<br>(-1%, 18%)	-5%*<br>(-10%, -1%)	-30%***<br>(-40%, -20%)	-98%***<br>(-118%, -78%)	-12%*<br>(-22%, -3%)
Short stay surgery/Gynae (Floor 6)	-35%***<br>(-40%, -31%)	-17%***<br>(-27%, -8%)	-10%***<br>(-14%, -5%)	-20%***<br>(-30%, -10%)	3%<br>(-17%, 23%)	-1%<br>(-11%, 8%)
Paediatrics (Floor 11)	0%<br>(-4%, 5%)	-1%<br>(-11%, 8%)	1%<br>(-4%, 5%)	4%<br>(-6%, 14%)	0%<br>(-20%, 20%)	15%**<br>(6%, 25%)
Adolescent (Floor 12)	16%***<br>(11%, 20%)	-2%<br>(-11%, 8%)	2%<br>(-3%, 6%)	-1%<br>(-11%, 9%)	-6%<br>(-26%, 14%)	4%<br>(-5%, 14%)
Oncology (Floor 13)	3%<br>(-2%, 7%)	-4%<br>(-13%, 6%)	1%<br>(-3%, 6%)	-14%**<br>(-23%, -4%)	-3%<br>(-23%, 17%)	1%<br>(-9%, 10%)
Head & Neck (Floor 14)	-6%**<br>(-11%, -2%)	-6%<br>(-15%, 4%)	-5%*<br>(-10%, -1%)	-7%<br>(-17%, 3%)	-7%<br>(-28%, 13%)	-3%<br>(-13%, 6%)
Private wards (Floor 15)	1%<br>(-4%, 5%)	13%**<br>(3%, 22%)	-11%***<br>(-15%, -6%)	-6%<br>(-16%, 4%)	-5%<br>(-26%, 15%)	39%***<br>(30%, 49%)
Haematology (Floor 16)	-4%<br>(-8%, 1%)	-3%<br>(-12%, 7%)	-2%<br>(-6%, 3%)	-7%<br>(-17%, 3%)	3%<br>(-17%, 23%)	-3%<br>(-13%, 7%)

*p < 0.05; **p < 0.01; ***p < 0.001

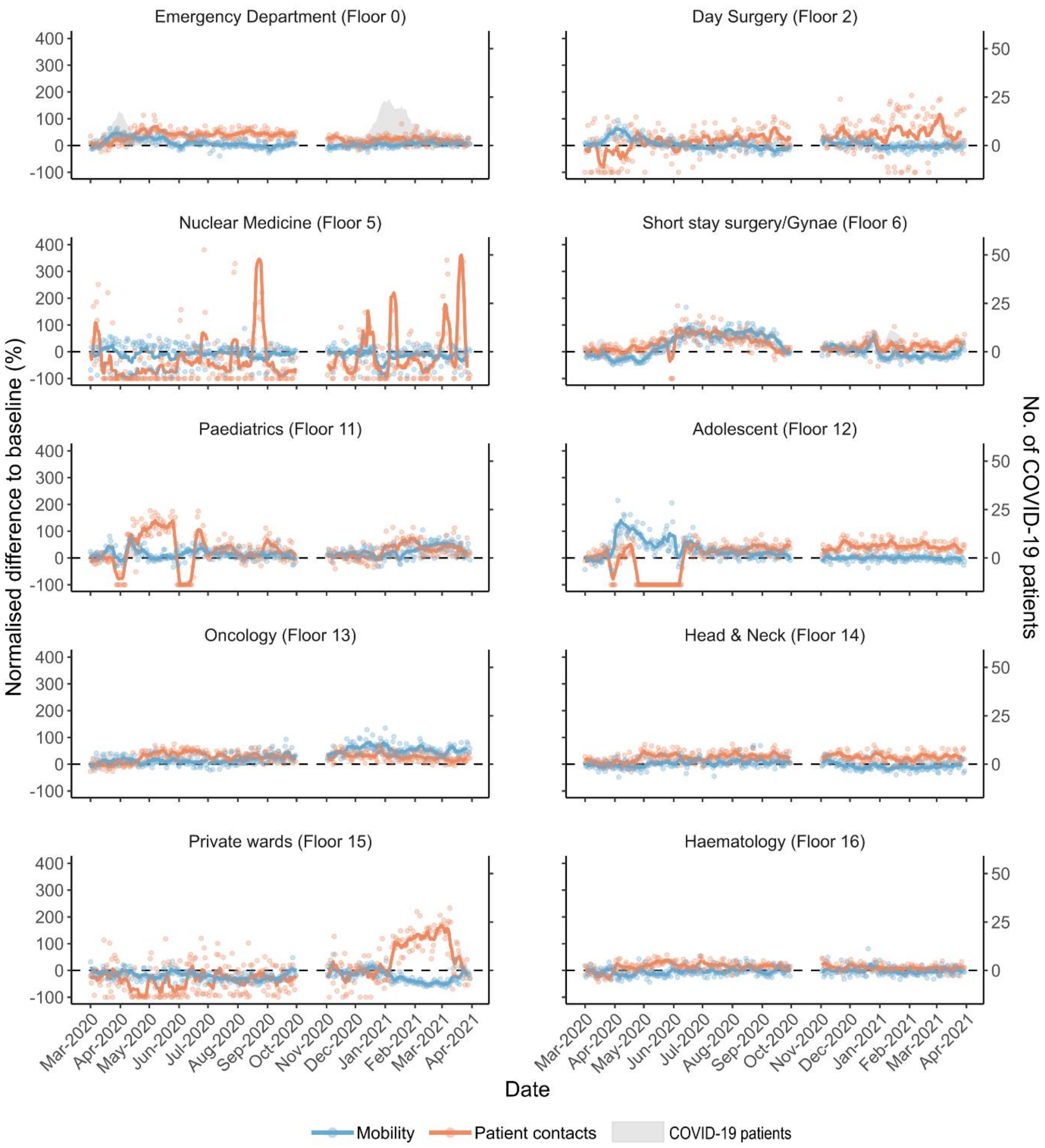

Figure S1. Changes in the daily rate of healthcare worker mobility and patient contacts on non COVID-19 floors in the Tower building at University College London Hospital during the first year of the COVID-19 pandemic. The normalized difference to baseline for daily rates of healthcare worker mobility (N_t^M; blue points) and patient contacts (N_t^C; red points) are plotted for non COVID-19 floors. The solid lines represent the seven day rolling averages. The black dotted line represents 0% change compared to the average in the pre-pandemic period. Non COVID-19 wards were identified as those that had <15% of all COVID-19 patients in the hospital during the observation period. The grey polygon highlights the number of COVID-19 patients on the floor. Data for October 2020 was not available.

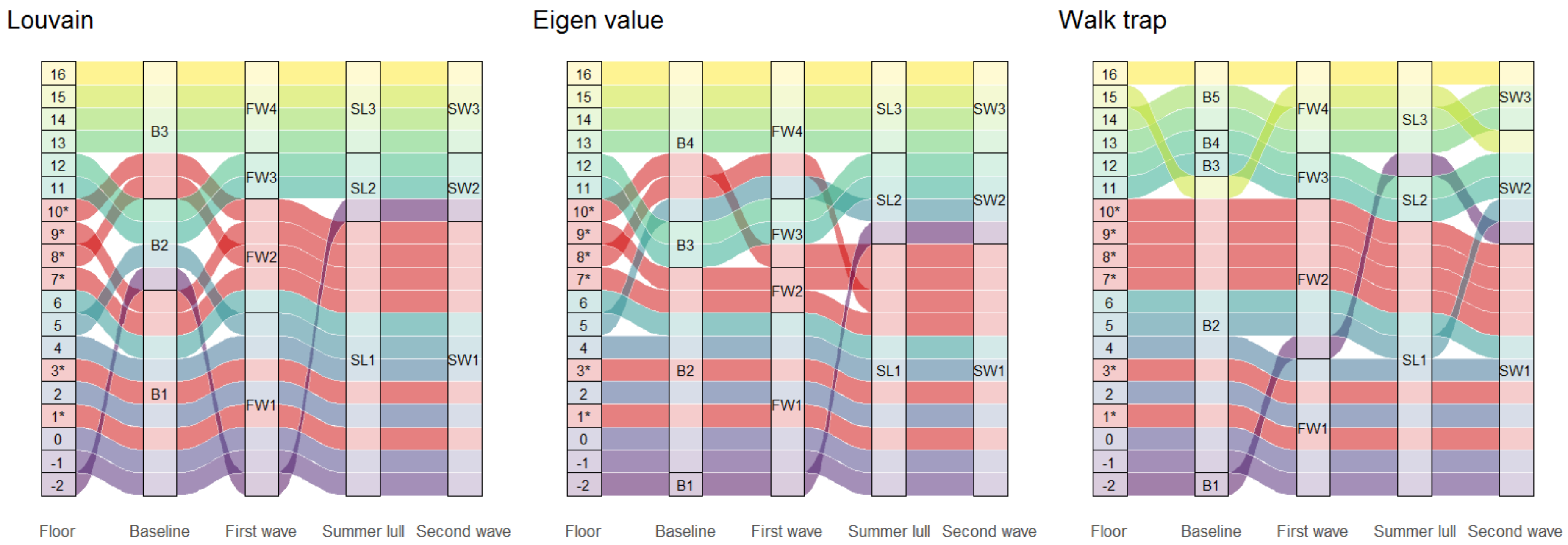

Figure S2: The spatial connectivity of floors in the Tower building at University College London Hospital during the COVID-19 pandemic. Each alluvial diagram depicts the connectivity of floors, as determined from either the Louvain clustering algorithm, leading eigenvector algorithm or random walk algorithm. Dyadic weights were derived from the number of healthcare workers with door events and/or patient contacts on the focal floors during the same day. The numbering in the left most column identifies the floors, and the codes in the remaining four columns represent the cluster group the floors belong to in each stage of the pandemic; pre-pandemic (baseline), first wave, summer lull and second wave. Floors that handled the majority (>=15%) of COVID-19 patients during the observation period are identified by an asterisk and their flows between clusters are in red.
