# Supplementary material for: Investigating healthcare worker mobility and patient contacts within a UK hospital during the COVID-19 pandemic
Source: Commun Med (Lond). 2022 Dec 23;2:165. doi: 10.1038/s43856-022-00229-x (PMC9782286; doi:10.1038/s43856-022-00229-x)
Supplement: Supplementary file 5 — Reporting Summary [file 43856_2022_229_MOESM5_ESM.pdf]

nature portfolio

Corresponding author(s): Ed Manley

Last updated by author(s): Nov 9, 2022

Reporting Summary

Nature Portfolio wishes to improve the reproducibility of the work that we publish. This form provides structure for consistency and transparency in reporting. For further information on Nature Portfolio policies, see our Editorial Policies and the Editorial Policy Checklist.

Statistics

For all statistical analyses, confirm that the following items are present in the figure legend, table legend, main text, or Methods section.

n/a	Confirmed	
☐	☒	The exact sample size (n) for each experimental group/condition, given as a discrete number and unit of measurement
☐	☒	A statement on whether measurements were taken from distinct samples or whether the same sample was measured repeatedly
☐	☒	The statistical test(s) used AND whether they are one- or two-sided<br>*Only common tests should be described solely by name; describe more complex techniques in the Methods section.*
☐	☒	A description of all covariates tested
☐	☒	A description of any assumptions or corrections, such as tests of normality and adjustment for multiple comparisons
☐	☒	A full description of the statistical parameters including central tendency (e.g. means) or other basic estimates (e.g. regression coefficient) AND variation (e.g. standard deviation) or associated estimates of uncertainty (e.g. confidence intervals)
☐	☒	For null hypothesis testing, the test statistic (e.g. F, t, r) with confidence intervals, effect sizes, degrees of freedom and P value noted<br>*Give P values as exact values whenever suitable.*
☒	☐	For Bayesian analysis, information on the choice of priors and Markov chain Monte Carlo settings
☐	☒	For hierarchical and complex designs, identification of the appropriate level for tests and full reporting of outcomes
☐	☒	Estimates of effect sizes (e.g. Cohen's d, Pearson's r), indicating how they were calculated

Our web collection on statistics for biologists contains articles on many of the points above.

Software and code

Policy information about availability of computer code

Data collection	Data were extracted from the databases at UCLH, no custom software was used.
Data analysis	Data analysis was conducted in R (v.4.0.0). Custom code used will be provided upon request.

For manuscripts utilizing custom algorithms or software that are central to the research but not yet described in published literature, software must be made available to editors and reviewers. We strongly encourage code deposition in a community repository (e.g. GitHub). See the Nature Portfolio guidelines for submitting code & software for further information.

Data

Policy information about availability of data

All manuscripts must include a data availability statement. This statement should provide the following information, where applicable:

- Accession codes, unique identifiers, or web links for publicly available datasets
- A description of any restrictions on data availability
- For clinical datasets or third party data, please ensure that the statement adheres to our policy

The data are available from UCLH but restrictions apply to protect the privacy of individuals, and so the data are not publicly available. Data are however available from the authors upon reasonable request and with permission from UCLH.

Human research participants

Policy information about studies involving human research participants and Sex and Gender in Research.

Reporting on sex and gender	This information was not collected
Population characteristics	See above
Recruitment	All members of staff at UCLH were enrolled in the study. All individual data were allocated against anonymised identifiers, and staff information posters and leaflets were disseminated to ensure awareness of the study. All analysis was conducted within the UCLH computer systems by UCLH staff and honorary staff, on the basis of 'legitimate interest'. A Data Protection Impact Assessment (DPIA) was carried out with the hospital to ensure these data were appropriately handled, and ethics approval granted on the basis of the research being an anonymised observation-based study.<br><br>Participation was dependent on whether healthcare workers logged a patient contact electronically or used their staff card to open a security door during the observation period. Differences in healthcare worker working patterns (i.e. how they use security doors and record patient contacts) could generate biases in the results. However, we present results at an aggregate level, and therefore do not expect such biases to generate significant impacts for the patterns described.
Ethics oversight	The study protocol was approved by the NHS Health Research Authority (South Central – Berkshire REC ref 20/SC/0147, protocol number 130861) and ethical oversight was provided by the UCLH research ethics committee (IRAS project ID: ref. 281836). UCL GOS ICH R&D approval number 20PL06.

Note that full information on the approval of the study protocol must also be provided in the manuscript.

Field-specific reporting

Please select the one below that is the best fit for your research. If you are not sure, read the appropriate sections before making your selection.

☐ Life sciences ☒ Behavioural & social sciences ☐ Ecological, evolutionary & environmental sciences

For a reference copy of the document with all sections, see nature.com/documents/nr-reporting-summary-flat.pdf

Behavioural & social sciences study design

All studies must disclose on these points even when the disclosure is negative.

Study description	A descriptive study using observational data retrospectively
Research sample	All healthcare workers in the UCLH Tower building
Sampling strategy	All healthcare workers with electronically recorded door access logs or patient contacts in the Tower building at UCLH during the observation period
Data collection	Data were extracted from routinely collected data sources at UCLH. Door access logs were recorded when healthcare workers used staff cards to open security doors. Patient contact events were recorded electronically by staff.
Timing	Data were extracted between January 1st 2020 – March 31st 2021. Data for the month of October 2020, a weekend in July and another in November were discarded, as records either could not be extracted or had an unusually low number of events (indicating an issue with extraction).
Data exclusions	Data for events outside the UCLH Tower were excluded. Contact events in EMRs that did not require face to face contacts (e.g. 'Telephone' or 'Letter') were excluded. Door events with a rejected status were removed along with duplicate events in the same direction that were within 60 seconds of each other. Two types of lift (or elevator) events were present in the door access logs; 'Lift Calls' where a card is used to request a lift, and 'Lift commands' where a card is used before selecting which floor to go to. All 'Lift Call' events were removed as they overinflate the number of movement events
Non-participation	N/A
Randomization	N/A

Reporting for specific materials, systems and methods

We require information from authors about some types of materials, experimental systems and methods used in many studies. Here, indicate whether each material, system or method listed is relevant to your study. If you are not sure if a list item applies to your research, read the appropriate section before selecting a response.

Materials & experimental systems

n/a	Involved in the study
☒	☐ Antibodies
☒	☐ Eukaryotic cell lines
☒	☐ Palaeontology and archaeology
☒	☐ Animals and other organisms
☒	☐ Clinical data
☒	☐ Dual use research of concern

Methods

n/a	Involved in the study
☒	☐ ChIP-seq
☒	☐ Flow cytometry
☒	☐ MRI-based neuroimaging
